# Autumn nitrogen enrichment destabilizes ecosystem biomass production in a semiarid grassland

**DOI:** 10.1016/j.fmre.2022.08.014

**Published:** 2022-09-06

**Authors:** Yuqiu Zhang, Zhengru Ren, Haining Lu, Xu Chen, Ruoxuan Liu, Yunhai Zhang

**Affiliations:** a State Key Laboratory of Vegetation and Environmental Change, Institute of Botany, Chinese Academy of Sciences, Beijing 100093, China; b University of Chinese Academy of Sciences, Yuquan Road, Beijing 100049, China

**Keywords:** Biomass production, Community stability, Inner Mongolia, Seasonal nitrogen addition, Species asynchrony, Steppe, Variability

## Abstract

Nitrogen (N) deposition decreases the temporal stability of ecosystem aboveground biomass production (ecosystem stability). However, little is known about how the responses of ecosystem stability differ based on seasonal N enrichment. By adding N in autumn, winter, or growing season, from October 2014 to May 2020, in a temperate grassland in northern China, we found that only N addition in autumn resulted in a significantly positive correlation between ecosystem mean aboveground net primary productivity (ANPP) and its standard deviation and significantly reduced ecosystem stability. Autumn N-induced reduction in ecosystem stability was associated with the vanished negative effect of community-wide species asynchrony (asynchronous dynamics among populations to environmental perturbations) on the standard deviation of ecosystem ANPP in combination with the emerged positive effect of dominance (Simpson's dominance index that indicates the relative weight of dominant species in a community). Our findings indicate that autumn N addition might overestimate the negative effect of annual atmospheric N deposition on ecosystem stability, suggesting that to better evaluate the influence of N deposition in temperate grasslands, both field experiments and global modeling should consider not only the annual N load but also its seasonal dynamics. Moreover, further studies should pay more attention to the alteration in the ecosystem temporal deviations, which might be more sensitive to human-induced environmental changes.

## Highlights

• A six-year's seasonal N addition experiment was conducted in a temperate steppe.
• Only autumn N decreased the temporal stability of ecosystem primary productivity.
• Autumn N altered the effects of species asynchrony and dominance on stability.
• Temporal anomaly was a determinant of seasonal N-induced ecosystem stability loss.

## Graphical Abstract

**Figure:**
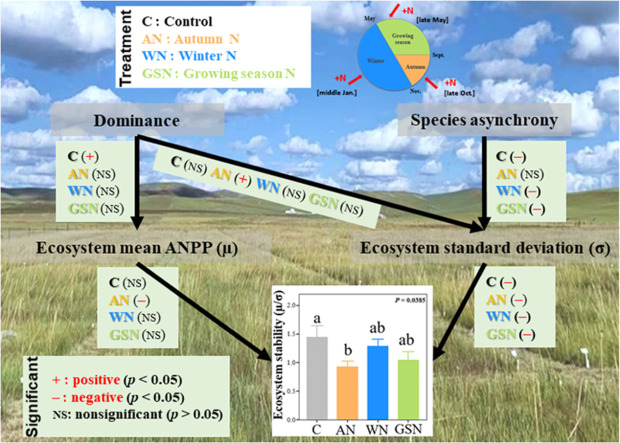

## 1 Introduction

Since the Industrial Revolution, there has been a marked increase in the emission of reactive nitrogen (N) from human activities, such as fossil fuel combustion, fertilizer overuse, and animal husbandry development [1], causing a considerable increase in global atmospheric N deposition. Nitrogen enrichment, both artificial and natural, can promote plant growth [2] and increase species dominance [3,4] in most terrestrial ecosystems. However, it also has adverse effects, such as decreasing plant diversity [5,6], altering community composition [7], and reducing ecosystem stability [8,9]. As ecosystem biomass production is vital for providing and predicting ecosystem functions and services [10], understanding the stability of ecosystem production is an immediate concern in the face of environmental changes [11]. Ecosystem stability, i.e., the temporal stability of ecosystem aboveground biomass production, can be calculated as the ratio of the temporal mean of ecosystem aboveground net primary productivity (ANPP) to its standard deviation [12]. Statistically, N-induced reduction in ecosystem stability can be indicated by a greater increase in the standard deviation of ecosystem ANPP than in the mean. N-induced reduction in ecosystem stability can be explained by the following mutually non-exclusive mechanisms, such as portfolio effect [12,13], compensatory effect [14,15], and sampling effect [16,17] (also referring to selection effect). N-induced plant diversity loss might lead to a direct reduction in ecosystem stability, which is associated with the portfolio effect [12,18], or indirectly via a decrease in species asynchrony (asynchronous dynamics among populations to environmental perturbations), which is related to the compensatory effect [19]. Species asynchrony can promote ecosystem stability via buffering temporal fluctuations in ecosystem productivity [20]. For example, N-induced reduction in the biomass of one or more species is likely to be compensated by the increase in the biomass of other species [21]. N-induced decrease in species asynchrony always causes a reduction in ecosystem stability, independent of species richness, which is related to the compensatory effect [21,22]. N addition may promote dominance of the relative biomass of N-favoring species at the community level [23,24], i.e., selection effect, possibly associated with a reduction in ecosystem stability [25]. This is because strong fluctuations in biomass are expected with interannual precipitation variations in grasslands [15]. Furthermore, N addition may reduce population stability which translates to ecosystem stability loss [9,21]. The above-mentioned mechanisms are based on results from simulating N deposition in field experiments through N addition during the growing season (but see Zhang et al., 2016 [9]). In other words, the mechanisms of seasonal N addition on ecosystem stability remain mostly unknown.

Notably, there is a traditional view on the seasonal distribution of atmospheric N deposition. Specifically, N deposition during the growing season is higher than that during the non-growing season, which is attributed to the seasonal intensity of human activities, such as N fertilization [26]. Nitrogen emissions from N fertilization are much higher in the growing season because of high temperatures [1]. The amount of N deposition in the non-growing season was less than that observed in the growing season possibly restricting plant growth. Therefore, many previous N deposition simulation studies have speculated that the effect of N deposition on plant growth during the non-growing season is negligible, resulting in an underestimation of the importance of N enrichment during the non-growing season. However, recent anthropogenic activities during the non-growing season, such as the burning of fossil fuels and the continuation and intensification of industrial reactive N emissions, have led to a continuous increase in atmospheric N deposition [26,27]. This has decreased the difference in the amount of N deposition between the growing and non-growing seasons. For example, the amount of atmospheric N deposition was similar throughout all seasons in eastern China over the past decade [27]. Seasonal N enrichment may increase soil N availability [28,29] which is often positively associated with ecosystem ANPP, affecting ecosystem stability in grasslands [30]. Thus, seasonal N enrichment may regulate the responses of ecosystem stability. On the one hand, N enrichment in the non-growing season may affect ecosystem N cycling in temperate ecosystems. For example, plants have equally high N uptake potential between growing and non-growing seasons in a temperate forest [31] and in a temperate heath ecosystem [32]. However, N uptake in plants after snowfall was markedly higher than that seen during the growing season in an alpine grassland ecosystem [33]. Conversely, N enrichment in the non-growing season can be partly retained by the ecosystem. For example, the balance of the temporal niche between plant and microbial activities can help a temperate grassland ecosystem fix N deposition in the non-growing season [34], which can be utilized for plant growth in the next growing season [35]. Temperate grassland ecosystems show apparent seasonal rhythms, and their plant community biomass production depends partially on the spatiotemporal availability of soil N [36]. Previous studies have shown that autumn N application stimulated leaching and denitrification, and N input during the growing season increased ammonia volatilization [37]. This means that winter N input would result in the smallest N emissions and the highest ecosystem productivity in the following year [37]. However, one study showed that plant community aboveground biomass in the following year did not greatly increase more by winter N addition than by spring N addition in a temperate grassland [28]. Furthermore, a recent study found that although the multi-year average ecosystem ANPP was also similar among seasonal N addition during the autumn, winter, or subsequent growing season, it was regulated by interannual precipitation [30]. This suggests that seasonal N additions may affect interannual variability of ecosystem ANPP through the magnitude of temporal standard deviation, which is an important regulating factor of ecosystem stability [8]. Therefore, the magnitude of negative effects on ecosystem stability among seasonal N inputs may not be equal. To date, few studies have investigated this issue, limiting our understanding of whether N addition in a single season can accurately assess the impacts of annual N deposition on ecosystems.

The temperate grassland in northern China contains a rich floral diversity and relatively low atmospheric N deposition [38], making it an ideal ecosystem to test the effects of N deposition on ecosystem stability. To assess these effects, we added N (10 g N m^–2^ year^–1^) in either autumn, winter, or the subsequent growing season, from 2014 to 2020, to compare the magnitude of the negative effects of N addition on ecosystem stability. Our previous results showed that although no considerable differences existed in the multi-year average ecosystem ANPP, species richness, and species composition among the three seasonal N additions, the addition of N in autumn and the growing season, rather than in winter, greatly improved the precipitation use efficiency [30]. Here, we hypothesized that: (1) autumn and growing season N additions, not winter N events, would considerably reduce ecosystem stability; and (2) the seasonal N-induced reduction in ecosystem stability is associated with the changes of species asynchrony, as species asynchrony plays a key role in promoting ecosystem stability in the grassland [9,39].

## 2 Materials and methods

### 2.1 Study site

The field experiment was conducted in a temperate steppe near the Inner Mongolia Grassland Ecosystem Research Station (IMGERS; 116°42′ E, 43°38′ N). The grassland is located in the Xilin River Basin, Inner Mongolia Autonomous Region, China. The experimental field has been fenced since 1999 to prevent grazing by large animals and is relatively flat, with an altitude ranging from 1259 to 1260 m above sea level. According to the long-term meteorological record (1984–2020), the mean annual temperature was 1.2 °C, with mean monthly temperatures ranging from −21.1 °C (January) to 20.1 °C (July). Following the customary guideline in the temperate grassland [15,40], one year includes three seasons, i.e., winter (last November to April), the growing season (May to August), and autumn (September to October). The mean annual precipitation was 344.1 mm, with approximately 71.2% falling during the growing season. The soil is classified as Haplic Calcisols and Calcic-Orthic Aridisol by the Food and Agriculture Organization of the United Nations and the USA soil classification system, respectively. Four perennial grasses, *Stipa grandis, Leymus chinensis, Achnatherum sibiricum*, and *Agropyron cristatum*, accounted for more than 87% of the community peak aboveground biomass production in the control during 2014–2020. The annual ambient atmospheric N deposition has remained < 1.0 g N m^–2^ in this region for the last four decades [38]. No fertilizer was applied before the experiment.

### 2.2 Experimental design

The field experiment was set up in July 2014 and followed a complete random block design [30]. We set four treatments: an ambient control and three seasonal N addition treatments (autumn in late October, winter without snow movement in mid-January, and the growing season in late May of the subsequent year). As differences in seasonal N deposition have been diminishing, i.e., the seasonal loads becoming similar at least in some regions [27], N as solid ammonium nitrate was evenly broadcasted at 10 g N m^−2^ year^−1^ (a widely used dose in evaluating the impacts of atmospheric N deposition in global grasslands) to evaluate the impacts of atmospheric N deposition in the temperate grassland. N additions in different seasons have different effects on N availability (Fig. S1). Each treatment was repeated eight times. Each experimental plot was 4 × 4 m, with a 1 m walkway between plots/blocks.

### 2.3 Field sampling

Plant community ANPP was estimated annually from peak aboveground plant biomass as all aboveground plant tissues die during the winter and there were no woody plants in the experimental field [41]. The peak aboveground plant biomass was sampled every year from 2014 to 2020 between 15 and 18 August using a 0.5 × 1 m strip, which was randomly placed in each 4 × 4 m experimental plot. The strips were placed at least 50 cm inside the border of each plot to avoid edge effects and had no spatial overlaps among the years. The aboveground components of all living vascular plants were cut at the soil surface, classified into species, dried in an oven at 65 ℃ for 48 h till they reached a constant weight, and then weighed. Species richness (the total number of species per 0.5 m^2^) was recorded every year in the same strip that ANPP was sampled in.

### 2.4 Statistical analyses

Ecosystem/species stability in each plot was quantified as the ratio of the temporal mean (μ) to its standard deviation (σ) of plant ANPP of community/species across the years 2015–2020 [42]. Ecosystem σ consists of the square root of summed variances of all species and the summed covariances of all pairs of species [42]. Population stability was calculated as the average of species stability across all species in the community.

Simpson's dominance index [43] in each plot every year was calculated as:

(1) $$\sum_{i=1}^{n}{\left(\frac{b_{i}}{B}\right)}^{2}$$

where *n* is the number of species in the sample, 1 ≤ *i* ≤ *n, b_i_* is the ANPP of species *i*, and *B* is the community ANPP in a plot. For each plot, Simpson's dominance index was averaged over 2015–2020.

Community-wide species asynchrony [14] was quantified as:

(2) $$1-\varphi_{b}=1-\sigma_{b_{T}}^{2}/{\left(\sum_{i=1}^{S}\sigma_{b_{i}}\right)}^{2}$$

where $\sigma_{b_{T}}^{2}$ is the variance of ecosystem ANPP and $\sigma_{b_{i}}$ is the standard deviation of ANPP of species *i* in a community with *n* species over the years 2015–2020. Species asynchrony ranges from 0 (perfect synchrony) to 1 (perfect asynchrony). A significant positive relationship between ecosystem stability and species asynchrony suggests that community-wide species asynchrony contributes to ecosystem stability [44].

To ensure normality and homogeneity, natural-logarithm transformation of the stability of ecosystem and population was performed before analyses. Linear mixed-effects models were used to assess the effects of seasonal N additions on ecosystem stability, ecosystem μ and σ, species richness, Simpson's dominance, population stability, species asynchrony, and the summed variances and covariances, using seasonal N as a fixed factor and block as a random factor. Tukey's test was used for *a posteriori* comparisons among the four treatments (α = 0.05).

The dissimilarity of plant species composition was assessed by a nonmetric multi-dimensional scaling (NMDS) ordination, based on Bray–Curtis distance measures, using the square root transformed plant species ANPP matrix (all stress < 0.2) each year (2015–2020). In addition, the effects of seasonal N additions on Bray–Curtis dissimilarity of plant communities each year (2015–2020) were evaluated using a one-way permutational multivariate analysis of variance (PERMANOVA; permutations = 999).

Simple linear regressions were applied to assess the relationships between species richness, Simpson's dominance, population stability, and species asynchrony and ecosystem stability. Linear regressions were also used to detect the relationship between ecosystem μ and its σ, thereby determining ecosystem stability. Since only Simpson's dominance and species synchrony were found to be significantly correlated with ecosystem stability, which is obtained by the ratio of ecosystem μ to its σ, we used simple linear regressions to analyze the relationships between ecosystem μ and its σ with Simpson's dominance and species synchrony. Similarly, linear regressions were employed to test the relationships between ecosystem μ and its σ and ecosystem stability, as well as the relationships between the summed variances (covariances) and ecosystem σ, Simpson's dominance, and species asynchrony.

All statistical analyses were performed on R version 4.1.2 [45], using the ‘nlme’ package for linear mixed-effects models, and the ‘ggplot2’ package for plotting all histograms and regression figures.

## 3 Results

### 3.1 Effects of seasonal N additions on ecosystem stability

**Fig. 1:**
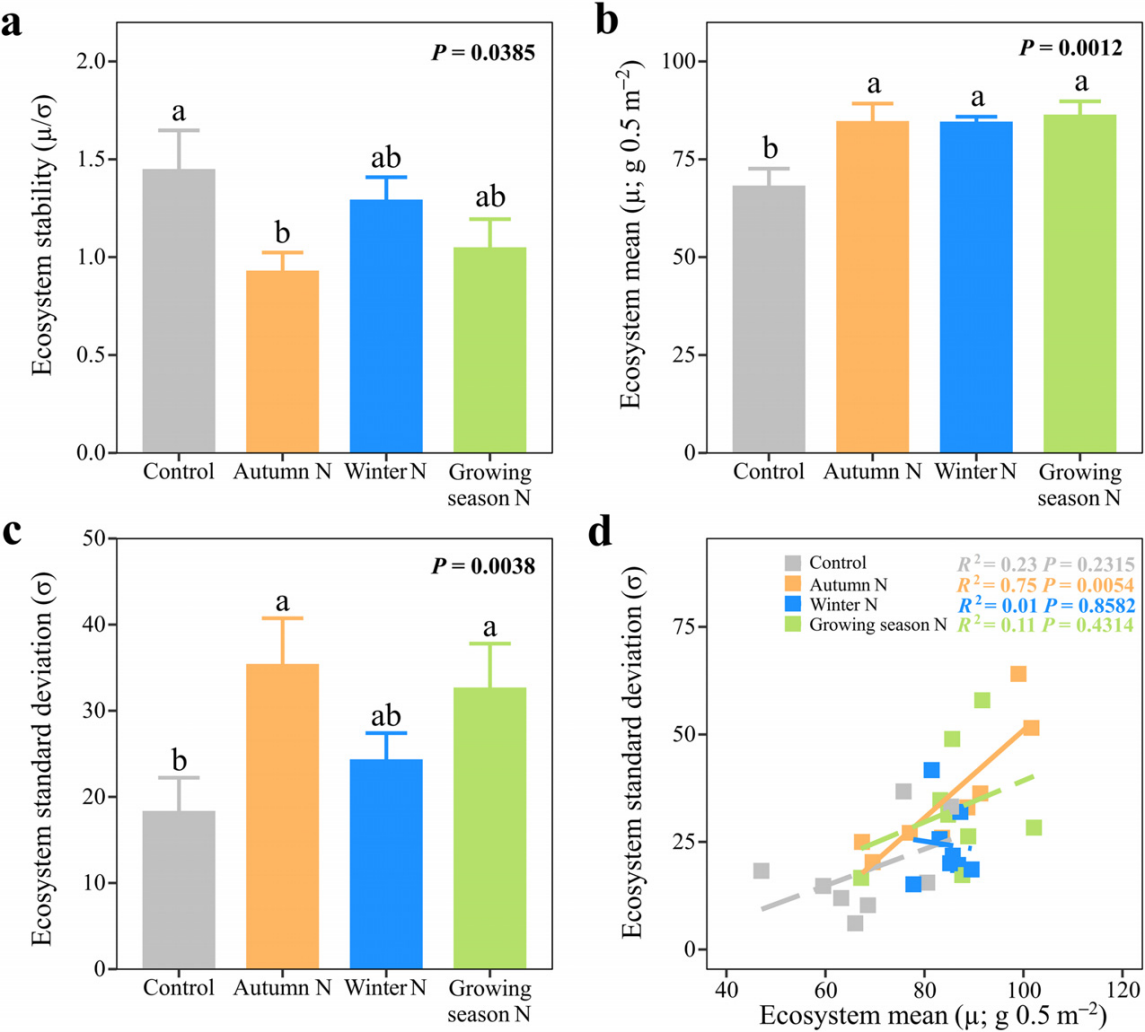
**Seasonal N additions and the temporal stability of ecosystem aboveground biomass production (ecosystem stability).** Effects of seasonal N additions on (a), ecosystem stability (μ/σ), (b), mean (μ) and (c), its standard deviation (σ) of ecosystem aboveground net primary productivity (ANPP) during the first six-treatment-year (2015–2020). (d), The relationships between ecosystem mean (μ) and its standard deviation (σ), respectively. Different letters indicate significant differences among the treatments (*P* < 0.05). Error bars indicate 1 SE (*n* = 8). In Fig. 1d, solid lines represent the significant regressions (*P* < 0.05).

Although ecosystem stability was non-significant among the three seasonal N additions, the addition of N in autumn significantly decreased ecosystem stability by 35.75% relative to that in the control (Fig. 1a; Table S1; *F*_3,21_ = 3.35, *P* = 0.0385). The ecosystem mean ANPP increased by 24.15%, 23.90%, and 26.54% under N addition during autumn, winter, and the growing season, respectively, relative to that in the control (Fig. 1b; Table S1; *F*_3,21_ = 7.73, *P* = 0.0012). The addition of N during autumn and the growing season significantly increased the standard deviation of ecosystem ANPP relative to that in the control by 93.01% and 78.06%, respectively (Fig. 1c; Table S1; *F*_3,21_ = 6.10, *P* =0.0038), though a non-significant difference was observed in the standard deviation of ecosystem ANPP among the three seasonal N additions. Ecosystem stability was significantly negatively associated with ecosystem mean ANPP with autumn N addition (*F*_1,6_ = 8.75, *P* = 0.0253) but not under the other three treatments (*P*s ≥ 0.7539). Ecosystem stability was negatively associated with ecosystem mean only under autumn N addition (Fig. S2a; *P* = 0.0253) and with ecosystem standard deviation in all four treatments (Fig. S2b; *P*s ≤ 0.0042). Moreover, a significant positive correlation between ecosystem mean ANPP and its standard deviation was seen only in cases of autumn N addition (Fig. 1d; *F*_1,6_ = 17.97, *P* = 0.0054).

### 3.2 Effects of seasonal N additions on species richness, dominance, population stability, and species asynchrony

**Fig. 2:**
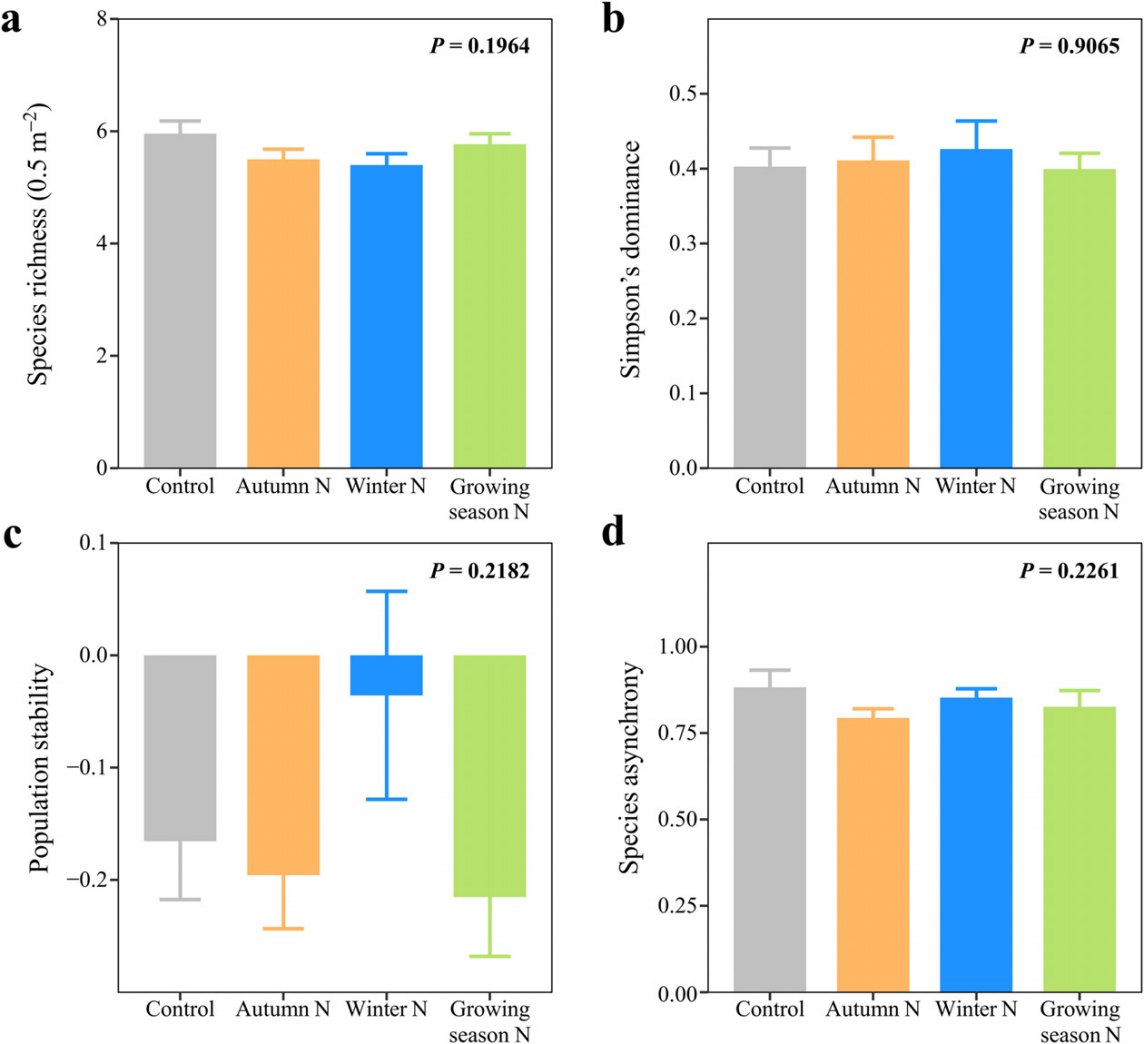
**Effects of seasonal N additions on species richness, Simpson's dominance, population stability, and species asynchrony.** Effects of seasonal N additions on (a), species richness (number of plant species 0.5 m^–2^), (b), dominance (the Simpson dominance index), (c), population stability, and (d), species asynchrony, respectively. Different letters indicate significant differences among the treatments (*P* < 0.05). Error bars indicate 1 SE (*n* = 8).

None of the seasonal N additions altered species richness (Fig. 2a; Table S1; *F*_3,21_ = 1.71, *P* = 0.1964), Simpson's dominance (Fig. 2b; Table S1; *F*_3,21_ = 0.18, *P* = 0.9065), population stability (Fig. 2c; Table S1; *F*_3,21_ = 1.61, *P* = 0.2182), species asynchrony (Fig. 2d; Table S1; *F*_3,21_ = 1.57, *P* = 0.2261), and the summed variances and covariances (Fig. S4; *P*s ≥ 0.2196). Plant species compositions were similar among all treatments according to the NMDS (Fig. S3; Table S2; PERMANOVA, *P*s ≥ 0.176).

### 3.3 Mechanisms of seasonal N additions affecting stability

Neither species richness (Fig. S5; *P*s ≥ 0.1939) nor population stability (Fig. S6; *P*s ≥ 0.2532) was correlated with ecosystem stability under any of the treatments. This suggests that seasonal N additions did not alter the relationships between species richness, population stability, and ecosystem stability. Moreover, no significant relationship between Simpson's dominance and species asynchrony was detected in any treatment (Fig. S7; *P*s ≥ 0.1915).

**Fig. 3:**
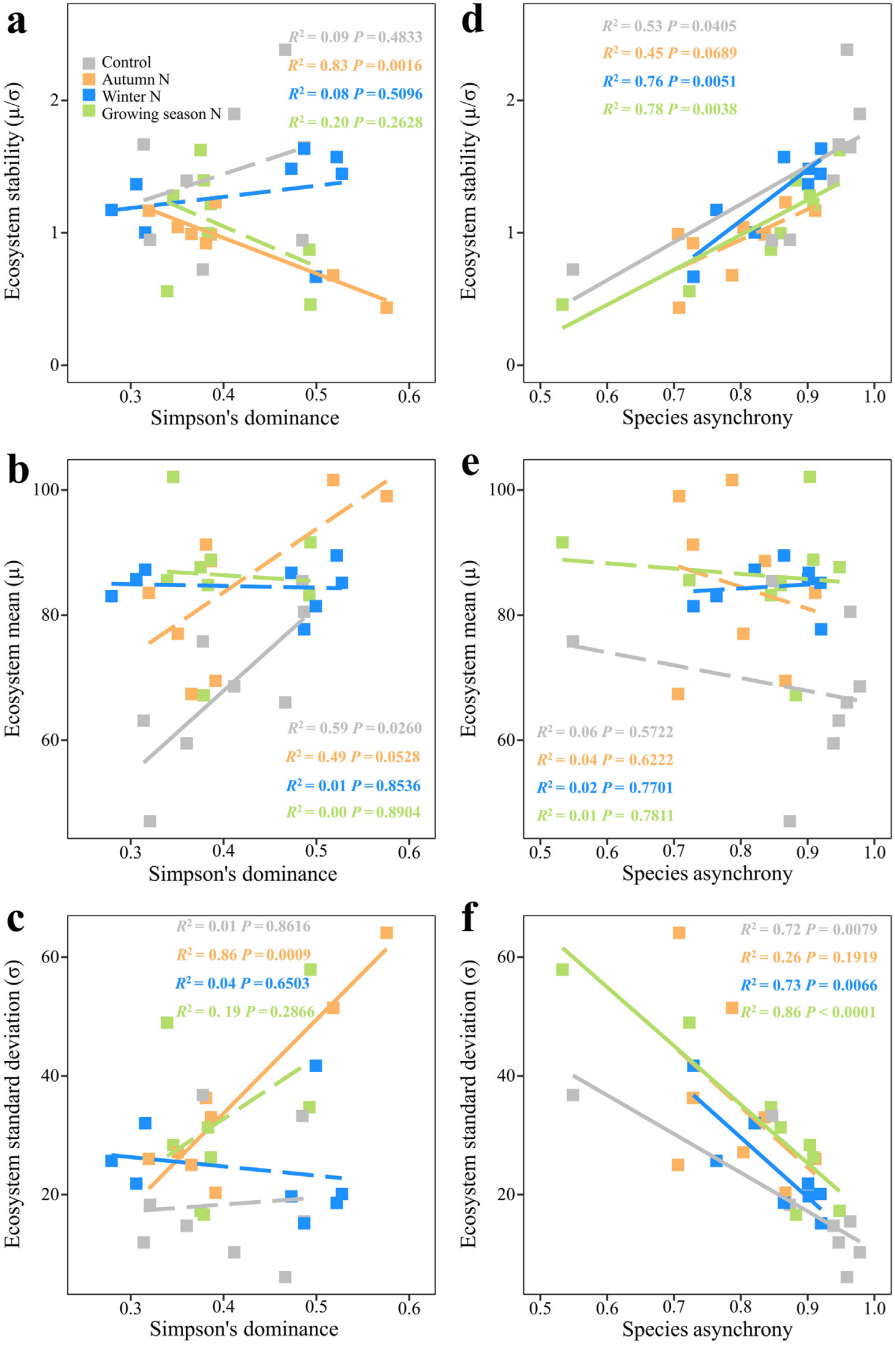
**Relationships between ecosystem stability and Simpson's dominance and species asynchrony.** Relationships between Simpson's dominance and (a), ecosystem stability, (b), mean and (c), its standard deviation of ecosystem ANPP, respectively. Relationships between species asynchrony and (d), ecosystem stability, (e), mean and (f), its standard deviation of ecosystem ANPP, respectively. The solid lines indicate significant regressions.

In cases of autumn N addition only Simpson's dominance negatively correlated with ecosystem stability (Fig. 3a; *F*_1,6_ = 29.53, *P* = 0.0016) but positively correlated with the standard deviation of ecosystem ANPP (Fig. 3c; *F*_1,6_ = 36.38, *P* = 0.0009). Simpson's dominance was positively correlated with ecosystem mean ANPP in the control (Fig. 3b; *F*_1,6_ = 8.64, *P* = 0.0260), but not in either seasonal N additions (Fig. 3b; *P*s ≥ 0.0528).

Except under autumn N addition (Fig. 3d and f; *P*s ≥ 0.0689), species asynchrony was positively correlated with ecosystem stability (Fig. 3d; *P*s ≤ 0.0405) but was negatively correlated with the standard deviation of ecosystem ANPP (Fig. 3f; *P*s ≤ 0.0079). No significant relationships between species asynchrony and ecosystem mean ANPP were detected in any of the treatments (Fig. 3d; *P*s ≥ 0.5722).

**Fig. 4:**
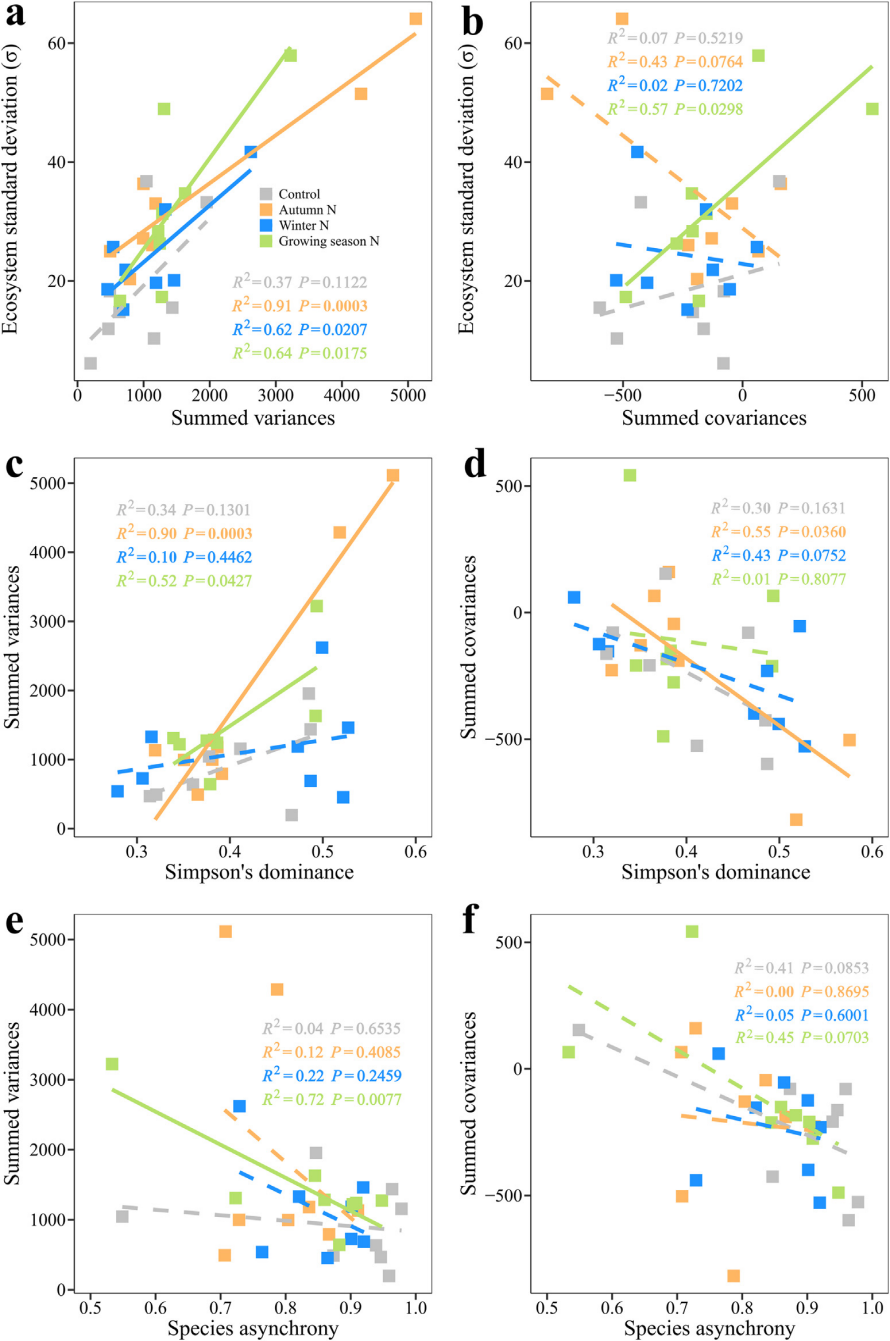
**Relationships between the summed variances (covariances) and ecosystem standard deviation, Simpson's dominance, and species asynchrony.** Relationships between ecosystem standard deviation and (a), the summed variances, (b), the summed covariances. Relationships between Simpson's dominance and (c), the summed variances, (d), the summed covariances. Relationships between species asynchrony and (e), the summed variances, (f), the summed covariances. The solid lines indicate significant regressions.

Ecosystem standard deviation was positively associated with the summed variances under all three seasonal N additions (Fig. 4a; *P*s ≤ 0.0207) and the summed covariances under the growing season N addition (Fig. 4b; *F*_1,6_ = 8.03, *P* = 0.0298). Moreover, Simpson's dominance was positively correlated with the summed variances under autumn N addition (Fig. 4c; *F*_1,6_ = 55.80, *P* = 0.0003) and the growing season N addition (Fig. 4c; *F*_1,6_ = 6.57, *P* = 0.0427), but was negatively correlated with the summed covariances only under autumn N addition (Fig. 4d; *F*_1,6_ = 7.24, *P* = 0.0360). Species asynchrony was negatively associated with the summed variances only under the growing season N addition (Fig. 4e; *F*_1,6_ = 15.45, *P* = 0.0077).

## 4 Discussion

By comparing the effects of seasonal N additions on ecosystem stability for six consecutive years from 2015–2020, we evaluated whether N addition in a single season can accurately assess the effects of annual N deposition. We found that though there was no significant difference among the effects of three of the seasonal N additions, only autumn N addition significantly reduced ecosystem stability relative to the control, partially supporting our first hypothesis. This suggests that adding N in the growing season (mostly in the simulated N deposition experiments conducted in temperate regions) might, to some extent, underestimate the negative effects of N deposition on ecosystem stability. To rephrase, the seasonal N inputs may mediate the effect of N deposition on ecosystem stability. Moreover, we found that though dominance and species asynchrony were similar among the seasonal N additions, autumn N-induced loss of ecosystem stability was caused by enhancing the positive effects of dominance and weakening the negative effects of species asynchrony on the standard deviation of ecosystem ANPP, partly supporting the second hypothesis. These results illustrate that the seasonal N-induced interannual anomalies in ecosystem biomass production were associated with both dominance and species asynchrony in regulating ecosystem stability.

### 4.1 Effects of seasonal N additions on species richness and asynchrony

It has been illustrated that N-induced multi-year average species richness loss is associated with experimental duration [6]. In our six-year N additions, species richness was not affected by seasonal N additions in the first four years, but was reduced in the following two years [30]. This resulted in seasonal N additions not showing a significant negative effect on multi-year average species richness. In addition, our previous study showed that a significant loss of species such as *Koeleria cristata* occurred after four years of experimental treatment [30]. This is consistent with the widespread negative effects of N enrichment on plant diversity in grasslands [6]. Previous studies have found that N enrichment increased the biomass of nitrophilous species by alleviating N limitations [46] but decreased that of dwarf species by increasing the competition for, and limitation of, light [5]. These processes cause isotropic responses in populations leading to an increase in species synchrony at the community level, which invariably decreases species asynchrony [9,22,47]. However, over our six-year-long experiment, we found that N addition did not alter species asynchrony, findings consistent with the results from a recent study of seven consecutive years of N addition in 34 grassland ecosystems worldwide [8]. This may be attributable to the unchanged relative biomass of dominant species (*S. grandis, L. chinensis, A. sibiricum*, and *A. cristatum* contribute to approximately 87% of the whole community) in the ecosystem of our study site [30]. Furthermore, we found that seasonal N additions did not alter species composition. Taken together, the lack of change in Simpson's dominance, population stability, and species asynchrony by seasonal N addition is reasonable. Therefore, future longer-term *in situ* experiments are needed to further verify these results.

### 4.2 Effects of seasonal N additions on ecosystem stability

We found that ecosystem stability was similar among the three seasonal N additions, but was significantly reduced only in the case of autumn N addition relative to that in the control. In this study, the temporal stability of ecosystem ANPP was calculated as the ratio of the temporal mean to its standard deviation over the six experimental years. Changes in ecosystem stability can result from the disproportionate changes in these two variables [8,9,18]. Previous studies mostly focused on exploring the effects of N enrichment on ecosystem mean ANPP and/or the correlation between ecosystem mean ANPP and its standard deviation [9]. Few studies investigated the degree of variation in the standard deviation of ecosystem ANPP (such as temporal or interannual anomalies) [8,18]. A recent global-scale study showed that N addition increased the temporal mean biomass production of grassland ecosystems by 21% and its standard deviation by 40%, thus reducing ecosystem stability [8]. Our study showed that both ecosystem mean ANPP and its standard deviation were not significantly different among the three seasonal N additions, but autumn N-induced reduction in ecosystem stability was associated with the intermediate increase in ecosystem mean ANPP (24.15%) and the highest increase in the standard deviation (93.01%). This was related to the mediation of precipitation on productivity as we recently found that the slope of the positive relationship between community ANPP and precipitation was the largest with autumn N addition in the same experimental platform [30]. In other words, autumn N addition can promote most plant community biomass production in wet, not dry years. This is caused by the N-induced difference in ecosystem ANPP between dry and wet years being the highest under N addition in autumn [30]. Meanwhile, ecosystem mean ANPP was significantly positively correlated with its standard deviation under autumn N addition, but not under the control, winter, and growing season N additions. These factors contributed to the lowest ratio of ecosystem mean ANPP to its standard deviation under autumn N addition. Our study suggests that ecosystem mean ANPP and its interannual anomalies are equally important when assessing the impacts of N addition on ecosystem stability. More importantly, as the seasonal amount of N deposition has become similar [27], our findings indicate that if we rely on the results from N addition in the growing season alone, we may underestimate the negative impacts of N deposition on ecosystem stability.

### 4.3 Mechanisms of seasonal N additions affecting ecosystem stability

Seasonal N additions did not alter the relationships between species richness, population stability, and ecosystem stability in this study. Autumn N addition resulted in a significantly negative correlation between Simpson's dominance and ecosystem stability, but decoupled the positive relationship between species asynchrony and ecosystem stability. Moreover, Simpson's dominance was positively associated with ecosystem mean ANPP under ambient conditions (the control), which is in line with previous studies [25]. Moreover, this positive relationship was not observed under either of the three seasonal N additions. Nitrogen enrichment always increases Simpson's dominance index [23,39] and interannual anomalies in ecosystem ANPP [18] and is dependent on the precipitation [30,48]. A positive correlation was observed between Simpson's dominance index and the standard deviation of ecosystem ANPP under N-enriched conditions. That is, a change in Simpson's dominance index under N addition is accompanied by a larger alteration in the interannual standard deviation of ecosystem ANPP. We found that this positive correlation was seen only in cases of autumn N addition. In our study area, ecosystem biomass production is limited by both water and N [48,49]. Furthermore, N addition in autumn and the growing season can considerably improve the precipitation use efficiency, that is, promoting more ecosystem ANPP during the wet years [30]. For example, in the year with the lowest productivity (2017), autumn N addition did not change ecosystem ANPP due to the scarce precipitation during the growing season, while in the year with the highest productivity (2018), it increased ecosystem ANPP by 91.83% due to the abundant precipitation during the growing season [30]. Therefore, autumn N addition caused the largest difference in ecosystem productivity between the two years, contributing to the largest standard deviation of ecosystem ANPP among the three seasonal N additions. Nitrogen added in autumn can provide more available N for perennial species to uptake and store belowground, which is associated with a greater increase in plant community aboveground biomass in the following wet, not dry years [27]. Autumn N addition tended to increase dominance and standard deviation in ecosystem ANPP in the same experimental plots, which in turn resulted in a positive correlation between dominance and ecosystem standard deviation. Moreover, our findings suggest that the enhanced correlation between Simpson's dominance and ecosystem standard deviation under autumn N addition was likely attributable to the positive effects of dominance on the summed variances in combination with the negative impacts on the summed covariances. Lehman and Tilman [42] reported that the summed variances can stabilize ecosystems when the increased biomass of individual species provides greater than its proportional increment in variance, while a smaller (i.e., more negative) summed covariances is often associated with a stronger competition and a more stable community. Accordingly, it could be implied that under autumn N addition, the negative effects of dominance on ecosystem standard deviation were due to the stronger positive effects on species variance than on its biomass in combination with the increases in interspecific competition, reducing ecosystem stability. In summary, although seasonal N additions did not change Simpson's dominance, autumn N addition resulted in a positive correlation between Simpson's dominance and standard deviation of ecosystem ANPP via the increases in species interannual variance and interspecific competition, which in turn reduced ecosystem stability.

Species asynchrony has also been widely demonstrated as an important determinant of ecosystem stability, that is, a greater species asynchrony is associated with higher ecosystem stability [20,50]. Species asynchrony promotes ecosystem stability through compensatory growth dynamics among species or functional groups [9,15]. A decline in ANPP of some species can be compensated by increases in ANPP of other species [30]. For example, the decrease in ANPP of *Cleistogenes squarrosa* and *K. cristata* was reported to be compensated by the increase of that in *S. grandis* and *L. chinensis* resulting in sustainable biomass production in our studied ecosystem [15]. We also found a significant positive correlation between species asynchrony and ecosystem stability under ambient conditions (the control), supporting the role of temporal compensatory dynamics among species for ecosystem stability [44]. This phenomenon was not always observed under seasonal N additions. Consistent with previous findings [9,47,50], species asynchrony promoted ecosystem stability under N addition in the growing season and winter. Conversely, a positive relationship between species asynchrony and ecosystem stability could not be detected under autumn N addition, thereby, weakening the positive relationship. This is attributable to the non-significant relationship between species asynchrony and the standard deviation of ecosystem ANPP. The weakened relationship might be associated with fewer changes in biomass-weighted species composition but with an increase in standard deviation in ecosystem ANPP under autumn N addition, relative to those in the growing season N input. Moreover, the diminished (i.e., insignificant) relationship between species asynchrony and ecosystem standard deviation under autumn N addition might be attributable to the non-significant relationships between species asynchrony and the summed variances and covariances. These results suggest that under autumn N addition, although species asynchrony did not affect either the summed variances (species interannual fluctuation) or covariances (interspecific competition), it negatively influenced their sum. Thus, autumn N addition did not change species asynchrony, but decoupled the effect of species asynchrony on the standard deviation of ecosystem ANPP via inter- and intraspecific interactions, which then reduced ecosystem stability.

Here we found two mechanisms for only autumn N addition reduced ecosystem stability, even though seasonal N addition did not alter dominance and species asynchrony. First, a significant negative relationship between dominance and ecosystem stability was detected under autumn N addition only. This is likely associated with the positive correlation between dominance and ecosystem standard deviation (denominator of ecosystem stability equation). Second, the significant positive relationship between species asynchrony and ecosystem stability was not detected under autumn N addition only. This is likely associated with the weakened (i.e., non-significant) correlation between species asynchrony and ecosystem standard deviation. Therefore, autumn N-induced reduction in ecosystem stability was attributable to alterations in the relationships between Simpson's dominance and species asynchrony and ecosystem standard deviation (denominator) rather than its mean (numerator). Variables that increase the interannual anomalies of ecosystem mean ANPP through N enrichment may influence ecosystem stability. Previous studies have not thoroughly investigated how dominance and/or species asynchrony affect standard deviation of ecosystem ANPP, as opposed to ecosystem mean ANPP [9,50]. It has been reported that ecosystem standard deviation consists of both summed variances and covariances which can indicate the magnitude of inter- and intraspecific interaction [42]. Our study found that dominance and species asynchrony influenced the summed variances and covariances which affected ecosystem standard deviation and ecosystem stability under seasonal N additions. Accordingly, dominance and species asynchrony will influence the magnitude of inter- and intraspecific interactions, then ecosystem standard deviation, and ultimately ecosystem stability. Therefore, this study provides solid evidence to uncover how N-induced loss of ecosystem stability is affected by the unaltered species asynchrony and dominance by N addition through changes in the magnitude of ecosystem standard deviation.

## 5 Conclusion

From the six consecutive years of *in situ* simulated N deposition experiments, we found that ecosystem stability, ecosystem mean ANPP, and its standard deviation were similar among three seasonal N additions, and only autumn N addition reduced ecosystem stability relative to that in the control. Nitrogen addition in autumn, rather than the traditional method of adding N in the growing season, caused a reduction in ecosystem stability. This suggests that ignoring the seasonal N dynamics of annual N deposition will lead to an underestimation of its negative effects on ecosystem stability. The autumn N-induced ecosystem stability loss was indicated by an increase in ecosystem standard deviation and the intermediate increase in ecosystem mean ANPP. More importantly, autumn N-induced ecosystem stability loss was associated with the emerged negative effect of dominance through a positive relationship between dominance and the standard deviation of ecosystem ANPP, and the weakened positive effect of species asynchrony through limiting the negative relationship between species asynchrony and standard deviation of ecosystem ANPP. Overall, our study highlights the importance of seasonal N dynamics to more accurately assess interannual/temporal anomalies in biomass production to better understand the mechanisms of atmospheric N deposition on ecosystem stability.

## Declaration of competing interest

The authors declare that they have no conflicts of interest in this work.

## Appendix Supplementary materials

Image, application 1

Image, application 2
