# Supplementary material for: Autumn nitrogen enrichment destabilizes ecosystem biomass production in a semiarid grassland
Source: Fundam Res. 2022 Sep 6;3(2):170–8. doi: 10.1016/j.fmre.2022.08.014 (PMC11197746; doi:10.1016/j.fmre.2022.08.014)

**Supplementary Materials**

**Autumn nitrogen enrichment destabilizes ecosystem biomass production in a semiarid grassland**

Yuqiu Zhang^a,b^, Zhengru Ren^a,b^, Haining Lu^a,b^, Xu Chen^a,b^, Ruoxuan Liu^a,b^, Yunhai Zhang^a,b,^*

*^a^State Key Laboratory of Vegetation and Environmental Change, Institute of Botany, Chinese Academy of Sciences, Beijing 100093, China*

*^b^University of Chinese Academy of Sciences, Yuquan Road, Beijing 100049, China*

** Corresponding author: zhangyh670@ibcas.ac.cn (Y. Zhang). Tel.: +86 10 62836581, Fax: +86 10 82596146.*

This supporting information contains 12-pages, including 7 figures, 2 tables, and this cover page.

**Table S1** Results of analysis of linear mixed-effects models for the effects of seasonal N additions on ecosystem temporal stability, ecosystem mean (μ) and its standard deviation (σ), species richness, Simpson's dominance, population stability, and species asynchrony during the experiment period (2015–2020). Degrees of freedom (*df*), *F*-test values and *P*-values are given.

|  | *df* | *F*-value | *P*-value |
| --- | --- | --- | --- |
| Ecosystem stability | 3,21 | 3.35 | 0.0385 |
| Ecosystem μ | 3,21 | 7.73 | 0.0012 |
| Ecosystem σ | 3,21 | 6.10 | 0.0038 |
| Species richness | 3,21 | 1.71 | 0.1964 |
| Simpson's dominance | 3,21 | 0.18 | 0.9065 |
| Population stability | 3,21 | 1.61 | 0.2182 |
| Species asynchrony | 3,21 | 1.57 | 0.2261 |

**Table S2** Results of one-way PERMANOVA testing the effects of seasonal nitrogen (N) treatments on Bray-Curtis dissimilarity of plant communities each year (2015–2020). Adonis2 function in vegan package in R (permutations = 999). Degrees of freedom (*df*), Sums of squares (SS), Mean squares (MS), *F*-, *R^2^* and *P*- values were given.

| Years | Source | *df* | SS | MS | *F* | *R^2^* | *P* |
| --- | --- | --- | --- | --- | --- | --- | --- |
| 2015 | N | 3 | 0.308 | 0.103 | 0.673 | 0.067 | 0.707 |
|  | Residual | 28 | 4.267 | 0.152 |  | 0.933 |  |
|  | Total | 31 | 4.574 |  |  | 1 |  |
| 2016 | N | 3 | 0.332 | 0.111 | 0.648 | 0.065 | 0.784 |
|  | Residual | 28 | 4.790 | 0.171 |  | 0.935 |  |
|  | Total | 31 | 5.123 |  |  | 1 |  |
| 2017 | N | 3 | 0.624 | 0.208 | 1.413 | 0.131 | 0.176 |
|  | Residual | 28 | 4.122 | 0.147 |  | 0.869 |  |
|  | Total | 31 | 4.745 |  |  | 1 |  |
| 2018 | N | 3 | 0.532 | 0.177 | 1.037 | 0.100 | 0.436 |
|  | Residual | 28 | 4.783 | 0.171 |  | 0.900 |  |
|  | Total | 31 | 5.315 |  |  | 1 |  |
| 2019 | N | 3 | 0.561 | 0.187 | 1.116 | 0.107 | 0.324 |
|  | Residual | 28 | 4.692 | 0.168 |  | 0.893 |  |
|  | Total | 31 | 5.253 |  |  | 1 |  |
| 2020 | N | 3 | 0.437 | 0.146 | 0.756 | 0.075 | 0.674 |
|  | Residual | 28 | 5.399 | 0.193 |  | 0.925 |  |
|  | Total | 31 | 5.836 |  |  | 1 |  |

**Fig. S1** Effect of seasonal N additions on the top 10 cm soil inorganic N concentrations (mg kg–1 dry soil) in July 2020 and during May–October 2021. Significant differences are reported as *P* < 0.05; *, *P* < 0.05; **, *P* < 0.01; ***, *P* < 0.001. Error bars indicate 1 SE (n = 8).

**Fig. S2** Relationships between ecosystem stability and ecosystem mean (μ) and ecosystem standard deviation (σ). Solid lines represent the significant regressions (*P* < 0.05).

**Fig. S3** Results of non-metric multidimensional scaling (NMDS) of plant communities across seasonal N addition from 2015 to 2020 (panels a–f, stress = 0.165, 0.143, 0.146, 0.133, 0.159, and 0.136, respectively; *P*s ≥ 0.176). NMDS was performed based on Bray-Curtis dissimilarity, which was measured with the square root transformed plant biomass of each species.

**Fig. S4** Seasonal N additions and the summed variances and covariances of species. Effects of seasonal N additions on (**a**), the summed variances, (**b**), the summed covariances. Error bars indicate 1 SE (n = 8).

**Fig. S5** Relationships between species richness and ecosystem stability. No significant regressions were detected in all treatments (all *P*s > 0.05).

**Fig. S6** Relationships between population stability and ecosystem stability. No significant regressions were detected in all treatments (all *P*s > 0.05).

**Fig. S7** Relationships between Simpson's dominance and species asynchrony. No significant regressions were detected in all treatments (all *P*s > 0.05).

**Fig. S1**


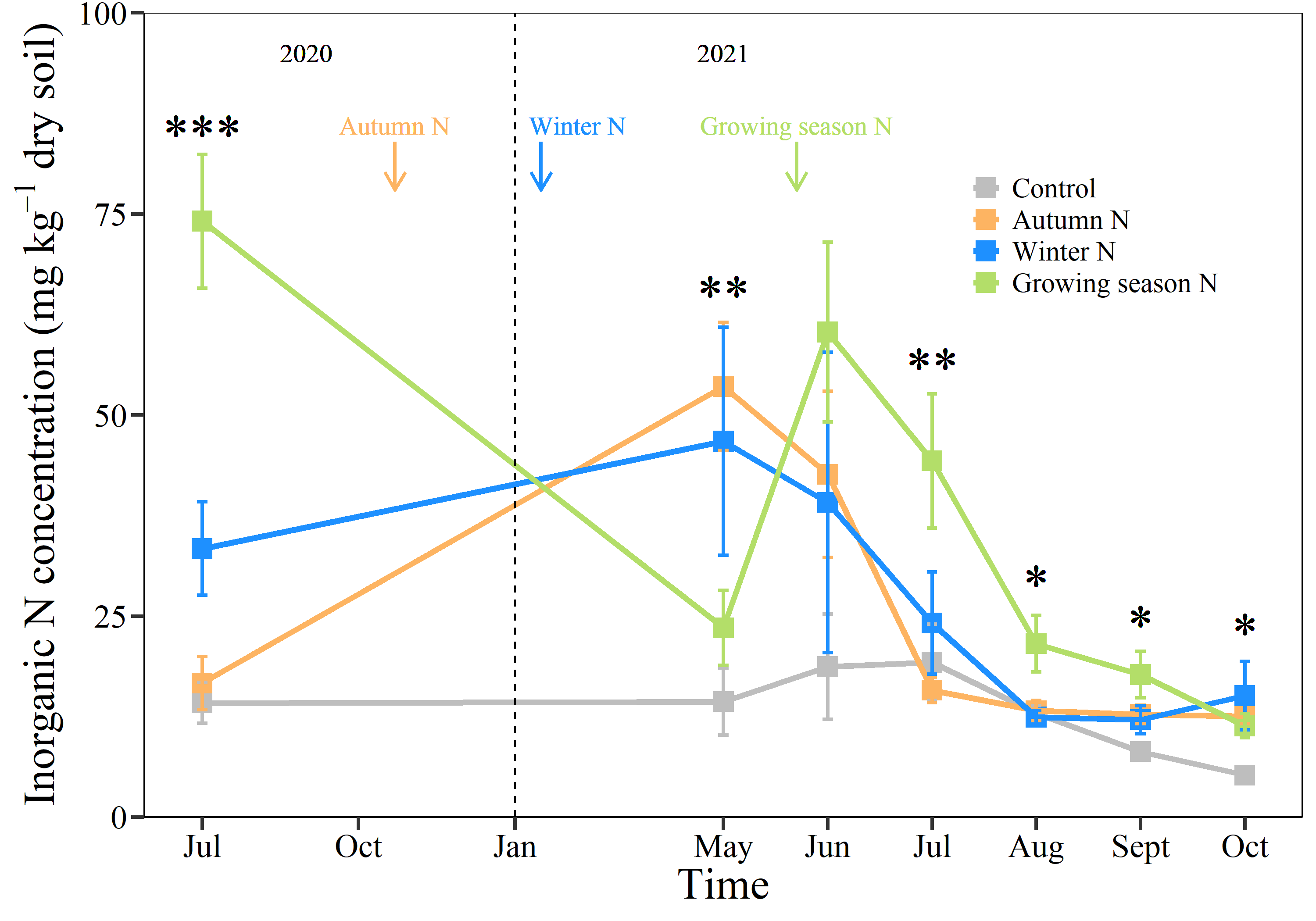


**Fig. S2**


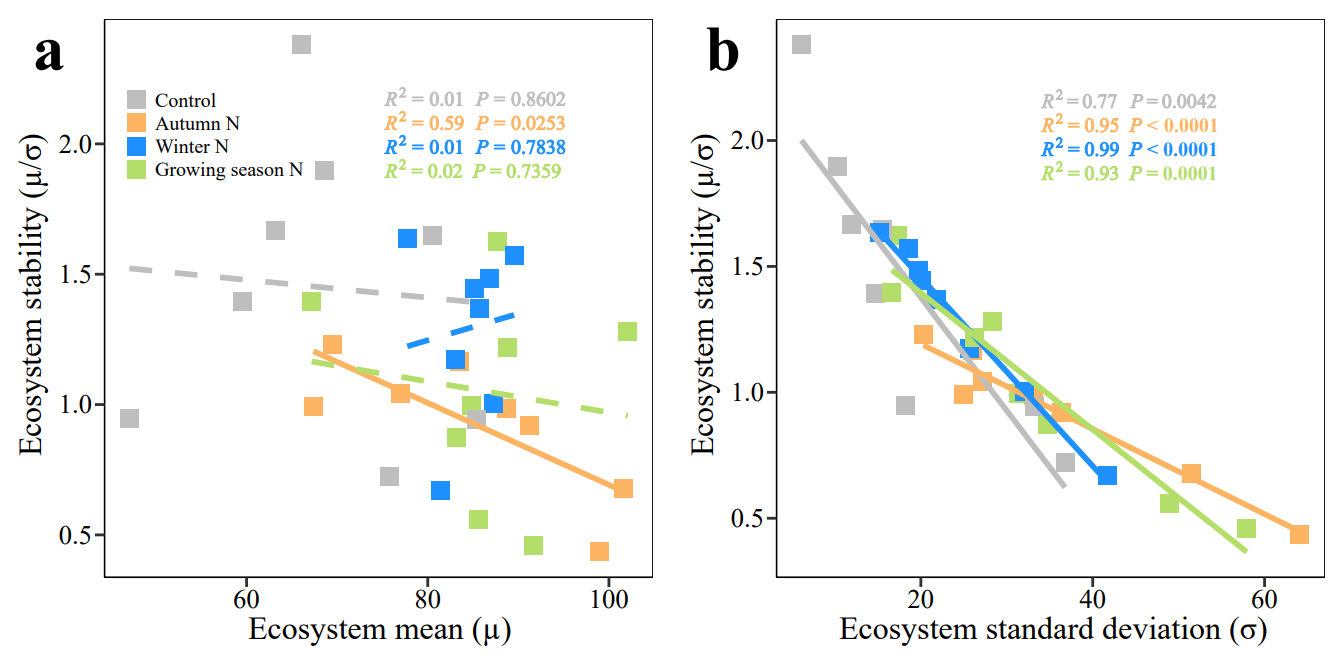


**Fig. S3**


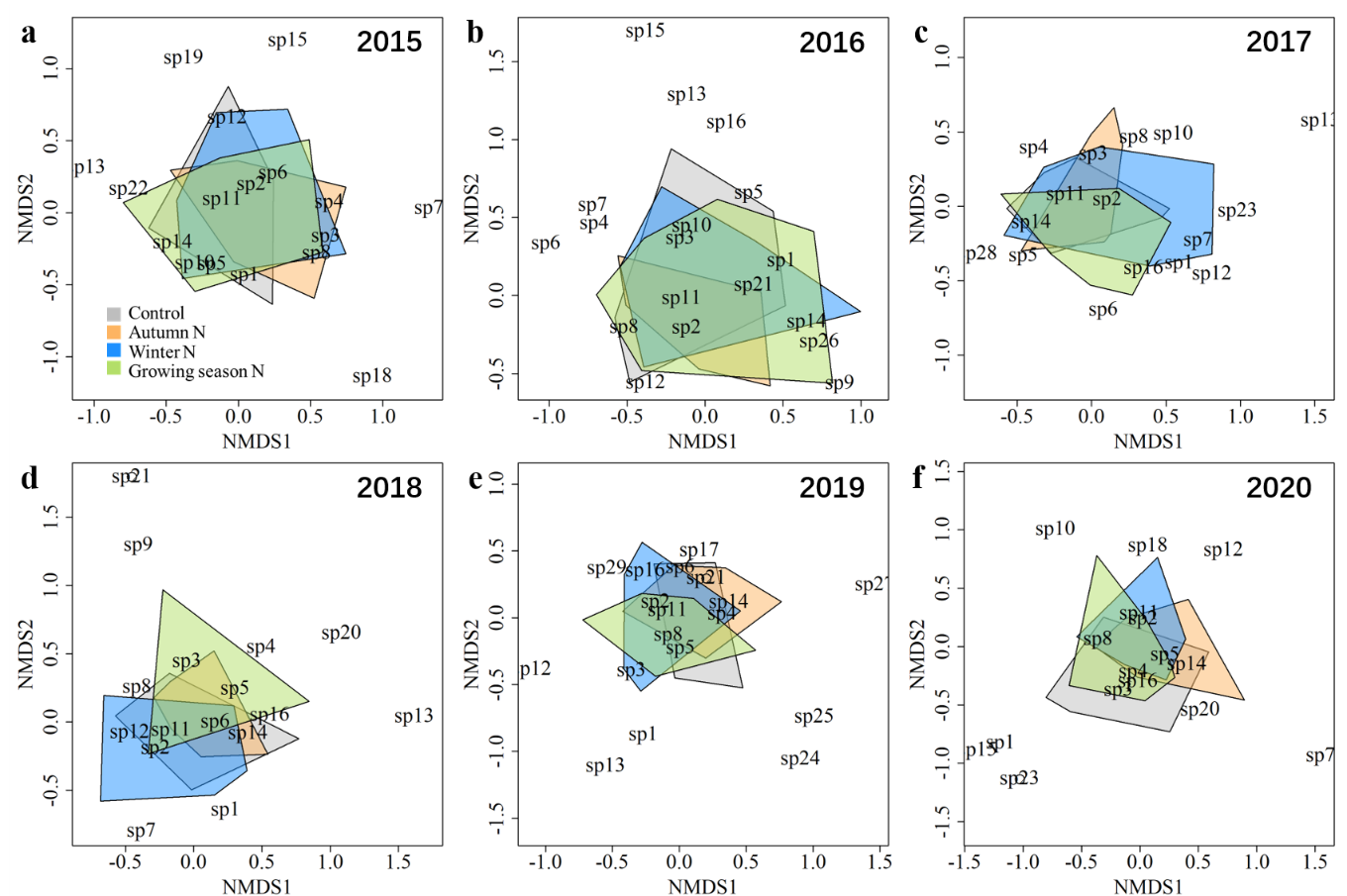


**Fig. S4**


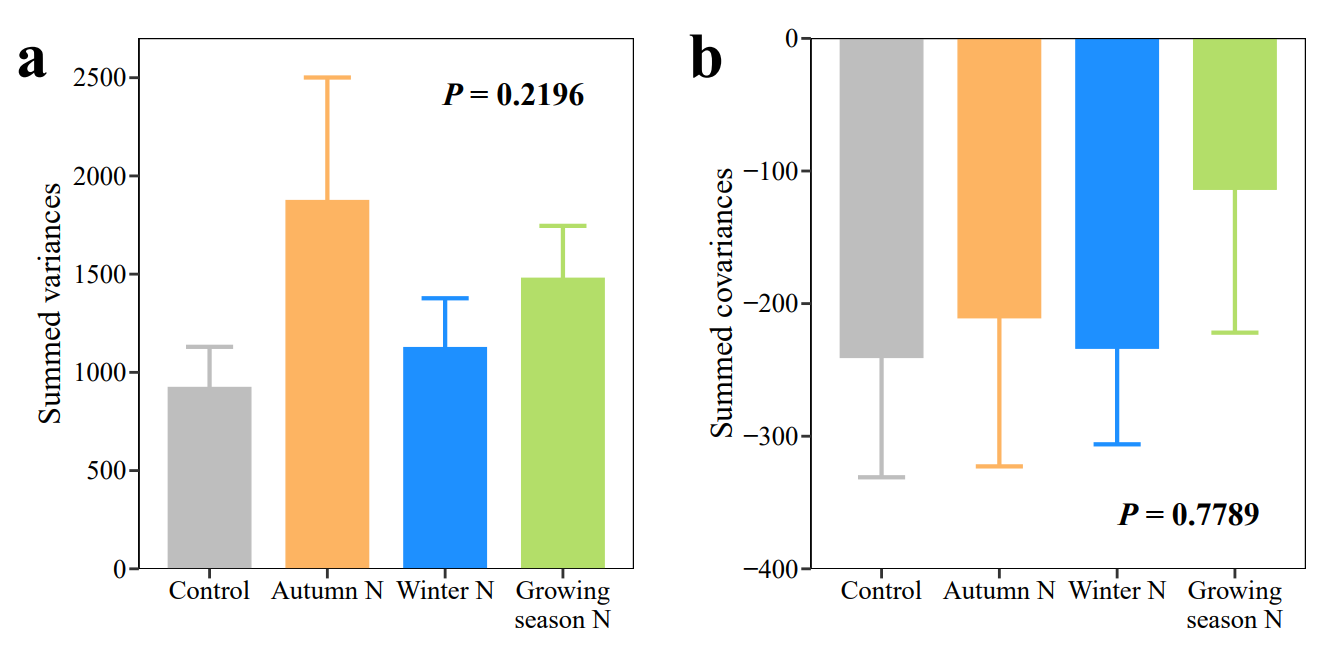


**Fig. S5**


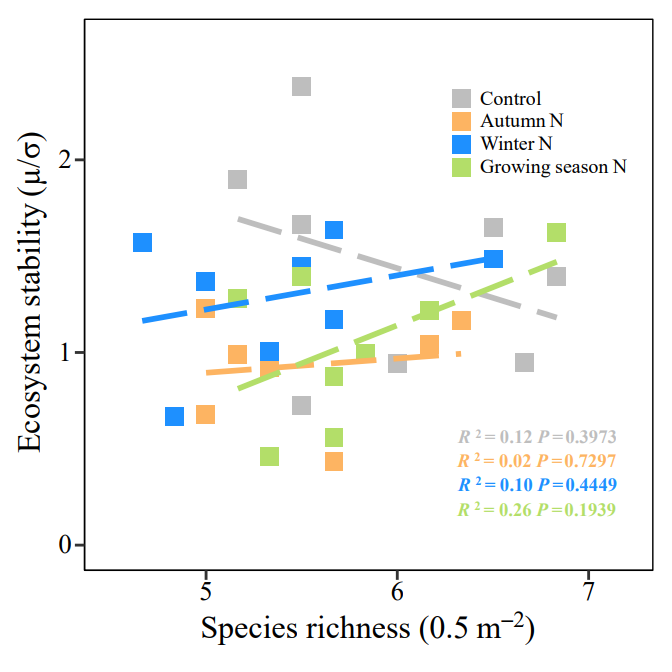


**Fig. S6**


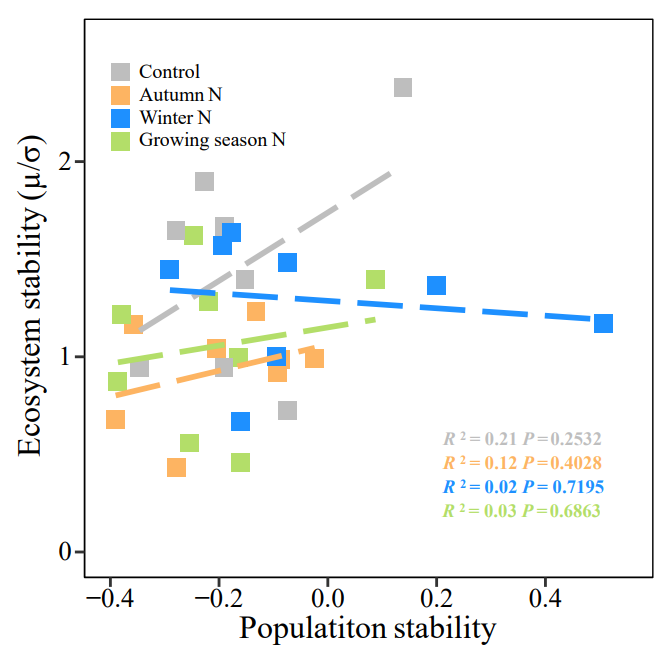


**Fig. S7**


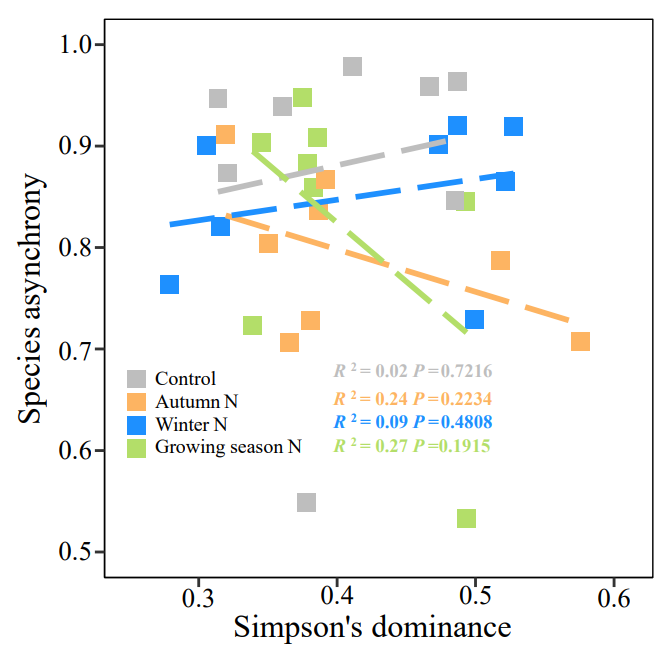

Supplement: Supplementary file 2 [file mmc2.docx]
